# The digital scalpel: navigating the future of surgical innovation

**DOI:** 10.1007/s00464-026-13131-7

**Published:** 2026-08-03

**Authors:** Derek T. O’Keeffe

**Affiliations:** University of Galway, Galway, Ireland

**Keywords:** Artificial intelligence, Surgical innovation, Digital surgery, Machine learning, Surgical training, Digital health equity, Intraoperative guidance

## Abstract

**Background:**

Artificial intelligence is entering surgical practice not as an extension of the surgeon's hand, eyes, or dexterity, but of surgical judgment itself. This creates a growing informational asymmetry between well-resourced and under-resourced operative environments, with implications for training, competence, accountability, and equity.

**Methods:**

This commentary, derived from the Karl Storz Lecture delivered at the SAGES 2026 Annual Meeting, proposes the Digital Scalpel: a five-layer conceptual architecture (data infrastructure, connected systems, computational vision, cognitive augmentation, and human judgment) for understanding AI-enabled surgical practice. The current evidence base for each layer is appraised, with particular reference to AI assessment of the Critical View of Safety in laparoscopic cholecystectomy.

**Results:**

Computational vision is the most mature layer, with deep-learning systems demonstrating high concordance with expert assessment of the Critical View of Safety, though validation remains largely confined to elective, uninflamed, single-centre cases. Three risks are identified: automation complacency, an unresolved accountability gap, and a digital inverse care law under which AI-augmented capability tracks existing resource inequities. The most evidence-supported entry point for most institutions is routine operative video capture and structured review, not real-time guidance.

**Conclusions:**

The surgeon of 2035 will require four attributes: preserved technical excellence, data literacy as a core competency, ethical fluency, and institutionalised clinician-engineer collaboration. The Digital Scalpel demands active, critical, and equitable engagement rather than passive technological adoption.

Every generation of surgeons has been defined by the tool it learned to master. The steel scalpel extended the surgeon’s hand, translating intention into incision. The laparoscope extended the surgeon’s eyes, taking vision inside the body. The surgical robot extended the surgeon’s dexterity, filtering tremor and enabling precision at a scale beyond unaided anatomy. Each of these transitions was disruptive. Each redefined what a surgeon was required to know, and what patients could expect.

We are now at the threshold of a fourth transition. Artificial intelligence is not extending the surgeon’s hand, eyes, or dexterity. It is extending something more fundamental: judgement. And that changes everything.

Consider two surgeons performing an identical laparoscopic cholecystectomy on the same day. The first works in an academic centre equipped with AI-powered intraoperative guidance, real-time tissue identification, anatomy probability heatmaps, and a predictive risk dashboard integrating the patient’s physiological and pharmacological data. The second works in a rural district hospital with a standard laparoscopic stack, good hands, and sound judgement—and nothing else. Same operation. Same procedure code. Profoundly different informational environments.

The difference between them is not skill. It is data—and data, in this context, does not merely inform: it shapes judgement. The first surgeon’s decisions are being made at the interface of human reasoning and machine intelligence. The second’s are not. That asymmetry is widening daily, and its implications touch every dimension of surgical practice: training, competence, accountability, and equity.

The integration of artificial intelligence into operative medicine has accelerated dramatically, driven by advances in computer vision, sensor miniaturisation, and cloud-based data infrastructure [1]. Surgery, once defined almost exclusively by tactile skill and anatomical knowledge, is rapidly becoming an information science—one in which data acquisition, interpretation, and real-time decision support are as consequential as the surgeon’s dexterity [2]. The incision is mechanical. The risk is informational.

The implications of this shift are profound. They touch on how we train surgeons, how we define competence, how we assign accountability, and how we ensure that the benefits of digital transformation do not accrue only to those operating in well-resourced environments. Access to AI-augmented care risks replicating existing structural inequities rather than resolving them [3].

This commentary introduces the digital scalpel—a conceptual and practical framework for understanding the architecture of AI-enabled surgical practice—and argues for a fundamental rethinking of surgical identity in the digital era.

## The digital scalpel stack

The term ‘digital scalpel’ is deliberately provocative. The scalpel is the most elemental of surgical instruments—precise, purposeful, and entirely subject to the surgeon’s will. To call the digital layer a scalpel is to assert that it carries equivalent weight: that commanding a data system is as consequential as making an incision.

**Fig. 1:**
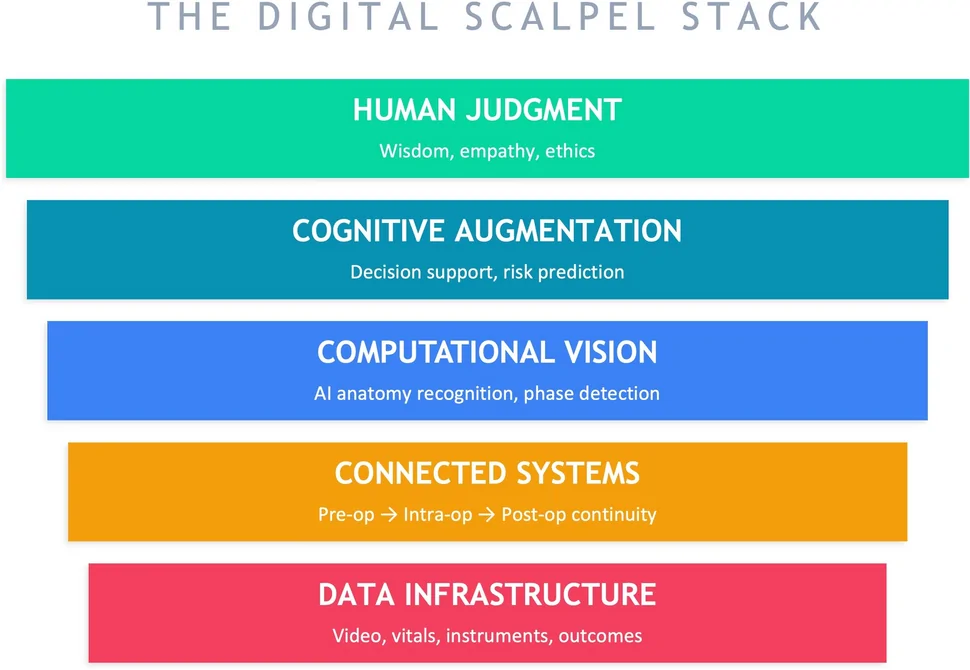
The digital scalpel stack. A five-layer architecture representing the integrated digital capabilities of AI-enabled surgical practice. Each layer depends on the integrity of the layer below it. Human judgement at the apex is informed by—but not subordinate to—the outputs of the underlying layers. Layer 1, data infrastructure: operative video, physiological monitoring, instrument telemetry, electronic health records, and outcome registries. Layer 2, connected systems: networked platforms enabling continuity of information across the surgical episode. Layer 3, computational vision: AI-enabled intraoperative image analysis, anatomical segmentation, and phase recognition. Layer 4, cognitive augmentation: predictive modelling and multi-modal decision support. Layer 5, human judgement: the irreducible apex—clinical wisdom, ethical reasoning, and operative accountability

The digital scalpel is best understood not as a single tool but as a five-layer architecture—a stack—each layer resting on the one below, and each amplifying or constraining what is possible above it (Fig. 1).

1. The foundation of the stack is the clinical data environment: operative video, physiological monitoring, instrument telemetry, electronic health records, and outcome registries. Without high-quality, structured, and interoperable data, no higher function in the stack is possible. Surgical data science as a discipline is premised on precisely this recognition—that the systematic capture and integration of perioperative data is a prerequisite for any intelligent application that follows [2]. Data is not merely an administrative byproduct of care; it is a clinical resource. As this author has stated in the surgical context: data is the new sterile field. If it is contaminated, everything downstream fails.

Layer 2—Connected systems The second layer concerns continuity of information across the surgical episode. The preoperative risk assessment, the intraoperative events, and the postoperative course are rarely integrated into a coherent data stream. Connected surgical systems—including networked instrument platforms, remote monitoring, and interoperable care pathways—enable the kind of longitudinal clinical intelligence that isolated data points cannot provide. The operation is no longer a discrete event: it is a data-inflection point in a continuous clinical record. Telesurgical platforms have already demonstrated the feasibility of transmitting operative capability across geographic boundaries, with systematic evidence now supporting their application in human subjects [4]. The implications for equity are significant: connectivity can in principle extend the informational environment of the academic centre to settings that have historically lacked it.

Layer 3—Computational vision The most mature application of AI in the operative domain is computer vision: the capacity of machine learning systems to analyse intraoperative video, identify anatomical structures, detect instrument–tissue interactions, and segment operative phases [5]. Tools now exist that can identify the safe and dangerous zones of dissection during laparoscopic cholecystectomy in real time, providing an additional layer of anatomical guidance that a skilled surgeon can interrogate, override, or act upon [6]. Humans see structure. Machines see probability. These systems do not replace surgical judgement; they extend what the operating surgeon can perceive.

Layer 4—Cognitive augmentation Above computational vision sits a more ambitious tier: AI systems that synthesise multiple data streams to provide decision support. Predictive models can now estimate intraoperative risk from preoperative data alone—including the anticipated operative difficulty of a cholecystectomy based on automated identification of gallbladder inflammation [7]. Large language models are beginning to be applied to operative documentation and postoperative note generation. The ambition of this layer is not to think for the surgeon, but to extend cognitive reach beyond what unaided human cognition can process in a complex operative environment. The surgeon is not replaced. The surgeon is amplified. Machines inform. Humans decide.

Layer 5—Human judgement The apex of the stack is, and must remain, the surgeon. It is the surgeon who integrates the outputs of all underlying layers—who decides when an AI flag is a true positive and when it reflects a limitation of the training data, who takes accountability for the incision, and who holds the patient’s welfare as the irreducible priority. Technology automates information; it does not automate wisdom. The stack is not a pyramid of ascending machine authority; it is an architecture designed to inform, extend, and protect human judgement.

## From concept to operating room: where the digital scalpel stands in 2026

How much of this can a surgeon use today? The five layers are at very different stages, and it is worth being plain about which are in the operating room now and which are still some way off.

Computational vision is the most developed layer, and the test that matters most to the laparoscopic surgeon is the critical view of safety. Deep-learning systems can now assess the Strasberg criteria from operative video. In a prospective series of elective cholecystectomies, an AI system agreed with blinded expert assessors on the view in every case [8]. In a separate study built into the laparoscopic video stack in real time, the model agreed with the operating surgeons whenever the view was achieved, and withheld confirmation in every case where inflammation or distorted anatomy meant it was never obtained [9]. Newer architectures classify the individual structures more finely again [10].

Three things temper this. First, almost all of the evidence comes from elective, single-centre, largely uninflamed cases—the very situations in which a good surgeon least needs help. The inflamed, scarred, hostile gallbladder that drives most bile duct injury is where these models are least tested and most likely to fail. An algorithm trained on elective gallbladders is a stranger in an inflamed one. Second, validation across institutions is still thin; doing well on one centre’s video says little about the next. The SAGES critical view of safety challenge, run across fifty-four institutions in 24 countries, exists to test whether these models generalise at all [11]. Third, no system is cleared anywhere to act as an autonomous arbiter of operative safety. For now these tools inform a decision; they do not make it.

So what should a surgeon in a general or rural hospital adopt? The most useful starting point is a modest one: the bottom of the stack, routine capture of operative video and its structured review. Commercial video platforms now let any hospital with a laparoscopic tower record, store, and analyse its own cases, which opens the door to objective postoperative review, coaching, and mentoring from an expert elsewhere. It is unglamorous beside a glowing overlay, but the evidence for benefit is strongest here and the cost of entry lowest. Where a real-time guidance tool is available, treat it as a checklist prompt and a second pair of eyes, and use it only within its tested range of elective, non-inflamed disease. And the best-validated way to clarify biliary anatomy in a remote theatre is not an algorithm but near-infrared fluorescence cholangiography, which sits alongside the computational layer rather than replacing it.

For the surgeon in a remote or under-resourced hospital, the message runs in two directions. The bedrock of safe cholecystectomy has not moved: sound technique, disciplined use of the critical view, and sensible recourse to cholangiography or fluorescence, none of which needs a data centre. At the same time, the AI most likely to reach that surgeon first is not the academic centre’s autonomous instrument but the plain pairing of video capture and remote review, the part of the field that could close the access gap rather than widen it. The digital inverse care law warns that the showiest tools arrive last where they are needed most. The answer is to put the modest, portable infrastructure first. A surgeon who records, reviews, and learns from each case is already doing digital surgery.

## Consequences and cautions

The integration of AI into surgical practice carries significant risks that must be confronted with the same rigour we apply to the technology itself.

### Automation complacency

Aviation offers a cautionary parallel. The introduction of autopilot systems dramatically improved flight safety, but also generated a well-documented phenomenon: automation complacency—the progressive degradation of manual skill and situational awareness in operators who rely heavily on automated systems. Research across aviation, nuclear power, and process control has demonstrated that complacency and automation bias represent different manifestations of the same underlying dynamic: when operators trust systems sufficiently, their capacity to detect and respond to system errors diminishes [12].

Surgery is not immune to this trajectory. As AI systems become more reliable, the temptation to defer to their outputs increases. The risk is not that surgeons will trust AI too little—it is that they will trust it too much, too uncritically, and without maintaining the independent capacity to operate when the system fails, misleads, or encounters a case outside its training distribution. If AI identifies the anatomy for us, do we lose the skill to do it ourselves? Training programmes must actively preserve foundational operative skills. The AI will not always be available.

### The accountability gap

Consider the following scenario: an AI system flags the critical view as unsafe and recommends conversion to open surgery. The operating surgeon disagrees, continues laparoscopically, and a common bile duct injury occurs. Alternatively, the AI declares the view safe; the surgeon proceeds; the same injury results. Who bears accountability in each case?

These questions do not yet have settled legal or ethical answers. What is clear is that accountability cannot be transferred to the machine. The surgeon who acts is the surgeon who is responsible. AI is a tool, and the craftsperson is accountable for the use of their tools. The profession must engage proactively with regulators, ethicists, and insurers to develop governance frameworks that are fit for this new environment—before adverse events force the issue [13].

### The equity imperative

The two surgeons described in the opening of this article inhabit the same professional world but not the same informational one. If access to AI-augmented surgical capability tracks existing patterns of resource distribution, the digital transformation of surgery will widen disparities rather than close them. This risk has a structural name: the digital inverse care law—the tendency for those who stand to benefit most from digital health innovation to be least likely to access it [3].

Addressing this requires deliberate policy choices: data infrastructure investment in underserved settings, open-source model development, and international collaboration on validation across diverse patient populations. It also requires surgeons to advocate for equity as a design criterion, not an afterthought.

## The surgeon of 2035

What does this transformation require of the surgeon? Four attributes seem essential.

### Technical excellence remains non-negotiable

The case for data literacy does not diminish the case for operative skill. AI systems fail. Networks go down. The surgeon who cannot operate without a dashboard is not a surgeon augmented by technology—they are dependent on it in a way that is dangerous. Manual competence is the foundation on which digital augmentation can safely rest.

### Data literacy must become a core surgical competency

Surgeons do not need to write code, but they do need to understand how AI systems are trained, validated, and liable to fail. They need to be able to critically appraise the performance characteristics of a system—its sensitivity, specificity, and the composition of its training dataset—with the same facility they bring to reading a randomised controlled trial. A surgeon who cannot interrogate the AI they are working with is in an analogous position to a surgeon who cannot read an anaesthetic chart.

## Ethical fluency is a new professional requirement

The questions raised by AI-assisted surgery—about accountability, consent, bias, and the nature of human judgement—are not questions that can be delegated to ethicists or regulators. They belong to the profession. Surgeons must be active participants in the governance of the technology they deploy [13].

### Clinician–engineer collaboration must be institutionalised

The gap between those who build surgical AI and those who use it remains dangerously wide. Bridging it requires not only interdisciplinary research teams but a new generation of surgeons trained to engage substantively with data science—to contribute to system design, challenge training datasets, and participate meaningfully in validation studies [14]. Surgical data science is not a subspeciality; it is a literacy that the entire profession needs.

## Conclusion

The scalpel extended our hands. The laparoscope extended our eyes. The robot extended our dexterity. The digital scalpel is extending our judgement—and that is the most consequential extension of all, because judgement is where the patient’s welfare ultimately resides.

The transformation underway is not a shift from craft to computation. It is a shift from craft to craft and computation—not replacing one with the other, but integrating both. The surgeon of the digital era must be technically excellent and data fluent, ethically grounded and technologically confident, deeply human and computationally literate.

The digital scalpel is not a metaphor for passive adoption of technology; it is a call to active, critical, and equitable engagement with the most significant transformation of operative medicine in a generation. Surgery has always asked its practitioners to be students of the tools they wield—to understand their limits as well as their capabilities, to take responsibility for how they are used, and to hold the patient’s welfare as the irreducible priority. That obligation does not change as the tools become more powerful. If anything, it becomes more important.

The digital scalpel cuts both ways. The profession’s task is to ensure that what it opens is opportunity—and what it closes is harm.
